# Sr–fresnoite determined from synchrotron X-ray powder diffraction data

**DOI:** 10.1107/S1600536812048921

**Published:** 2012-12-05

**Authors:** Anthony M. T. Bell, C. Michael B. Henderson

**Affiliations:** a HASYLAB/DESY, Notkestrasse 85, 22607 Hamburg, Germany; b School of Earth, Atmospheric and Environmental Sciences, University of Manchester, Manchester, M13 9PL, England

## Abstract

The fresnoite-type compound Sr_2_TiO(Si_2_O_7_), distrontium oxidotitanium disilicate, has been prepared by high-temperature solid-state synthesis. The results of a Rietveld refinement study, based on high-resolution synchrotron X-ray powder diffraction data, show that the title compound crystallizes in the space group *P*4*bm* and adopts the structure of other fresnoite-type mineral samples with general formula *A_2_*TiO(Si_2_O_7_) (*A* = alkaline earth metal cation). The structure consists of titanosilicate layers composed of corner-sharing SiO_4_ tetra­hedra (forming Si_2_O_7_ disilicate units) and TiO_5_ square-based pyramids. These layers extend parallel to the *ab* plane and are stacked along the *c* axis. Layers of distorted SrO_6_ octa­hedra lie between the titanosilicate layers. The Sr^2+^ ion, the SiO_4_ tetra­hedron and the bridging O atom of the disilicate unit are located on mirror planes whereas the TiO_5_ square-based pyramid is located on a fourfold rotation axis.

## Related literature

For the crystal chemistry of fresnoites, see: Barbar & Roy (2012 ▶); Höche *et al.* (2002 ▶); ICDD (1989 ▶). For properties of Sr–fresnoites, see: Park & Navrotsky (2010 ▶). Atomic coordinates as starting parameters for the Rietveld refinement (Rietveld, 1969 ▶) of the present phases were taken from Ochi (2006 ▶); Goldschmidt & Thomassen (1923 ▶); Machida *et al.* (1982 ▶); Mitchell *et al.* (2000 ▶). For related strontium titanosilicates, see: Miyajima *et al.* (2002 ▶). For synchrotron data analysis, see: Hammersley (1997 ▶); Hammersley *et al.* (1996 ▶).

## Experimental

### 

#### Crystal data

Sr_2_TiSi_2_O_8_
*M* *_r_* = 407.31
Tetragonal, 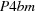

*a* = 8.3200 (3) Å
*c* = 5.0239 (2) Å
*V* = 347.77 (2) Å^3^
*Z* = 2
Synchrotron radiation, λ = 0.207549 Å
μ = 0.43 mm^−1^
*T* = 293 K
Cylinder, 20 × 0.7 mm

#### Data collection

In-house design diffractometer
Specimen mounting: capillary
Data collection mode: transmission
Scan method: continuous
2θ_min_ = 0.053°, 2θ_max_ = 11.915°, 2θ_step_ = 0.008°

#### Refinement

*R*_p_ = 0.052
*R*_wp_ = 0.073
*R*_exp_ = 0.031
*R*_Bragg_ = 0.093
χ^2^ = 31.068
1476 data points
71 parameters
5 restraints

### 

Data collection: local software; cell refinement: local software; data reduction: local software; program(s) used to solve structure: coordinates taken from a related compound; program(s) used to refine structure: *FULLPROF* (Rodriguez-Carvajal, 2001 ▶); molecular graphics: *VESTA* (Momma & Izumi, 2008 ▶); software used to prepare material for publication: *publCIF* (Westrip, 2010 ▶).

## Supplementary Material

Crystal structure: contains datablock(s) global, I. DOI: 10.1107/S1600536812048921/wm2699sup1.cif

Rietveld powder data: contains datablock(s) I. DOI: 10.1107/S1600536812048921/wm2699Isup2.rtv

Additional supplementary materials:  crystallographic information; 3D view; checkCIF report

## Figures and Tables

**Fig. 1.**
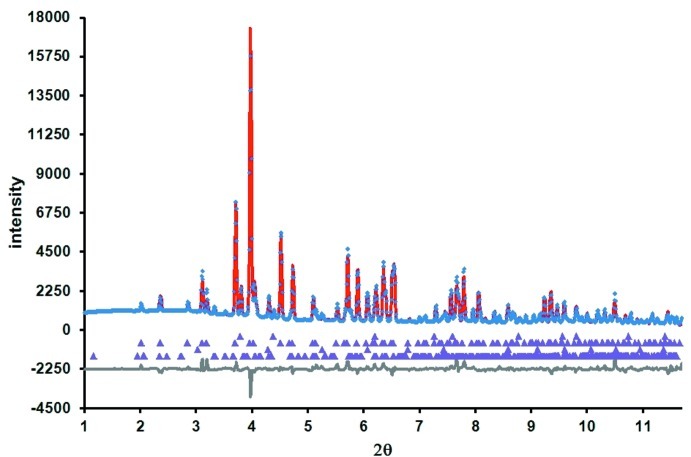
Rietveld difference plot for the multi-phase refinement of Sr2TiO(Si2O7), CeO2, SrTiO3 and SrSiO3. The blue crosses, and red and black lines show respectively the observed, calculated and difference plots. Calculated Bragg reflection positions are indicated by triangles for the four phases.

**Fig. 2.**
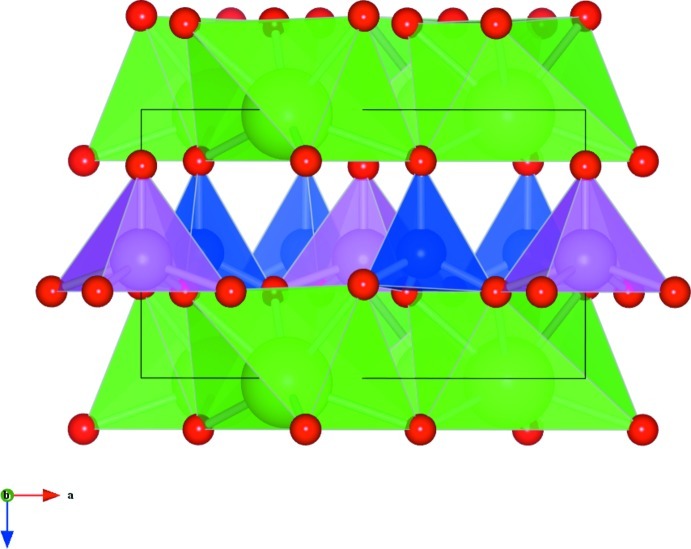
The crystal structure of Sr2TiO(Si2O7). Purple polyhedra show TiO5 units, blue polyhedra show SiO4 units, green polyhedra show distorted SrO6 units. Green spheres represent Sr atoms, pink spheres represent Ti atoms, blue spheres represent Si atoms and red spheres represent O atoms.

## supplementary crystallographic information

### Comment

The title compiound, Sr_2_TiO(Si_2_O_7_), is the Sr analogue of the mineral fresnoite, Ba_2_TiO(Si_2_O_7_). It is of interest as a potential storage medium for radioactive strontium from nuclear waste (Park & Navrotsky, 2010). An incommensurately modulated structure of Sr-fresnoite has been determined from room-temperature single crystal data and refined in the 5D superspace group *P*4*bm* (-α, α, 1/2; α, α, 1/2) with α = 0.3 and with lattice parameters *a* = 8.312 (2) Å and *c* = 10.07 (1) Å (Höche *et al.*, 2002). However, ICDD PDF card 39–228 (ICDD, 1989) states that at room temperature this material is tetragonal with space group *P*4*bm* and lattice parameters *a* = 8.3218 (2) Å and *c* = 5.0292 (2) Å. The crystal structure of the mineral fresnoite has been decribed in the same space group with lattice parameters *a* = 8.5159 (6) Å and *c* = 5.2184 (4) Å. Solid solutions with composition Ba_2-_*_x_*Ca*_x_*TiO(Si_2_O_7_) (*x* = 0.0, 0.2, 0.4, 0.8, 1.0; Barbar & Roy, 2012) adopt the same structure. The ordered crystal structure of Sr_2_TiO(Si_2_O_7_) in space group *P*4*bm* and a halved *c* parameter in comparison with the single crystal study is reported in the present communication.

The mean Si—O and Ti—O distances in the titanosilicate layer of Sr-fresnoite are respectively 1.64 Å and 1.92 Å. The corresponding Si—O and Ti—O distances are 1.64 Å and 1.93 Å in fresnoite. The respective distances in the structures of the solid solutions Ba_2-_*_x_*Ca*_x_*TiO(Si_2_O_7_) are: 1.59 Å and 2.01 Å (*x* = 0.2); 1.65 Å and 2.03 Å (*x* = 0.4); 1.66 Å and 2.02 Å (*x* = 0.8); 1.71 Å and 1.95 Å (*x* = 1.0) (Barbar & Roy, 2012). Due to the distortion of the crystal structures by the partial replacement of Ba by Ca these distances are less comparible with those in Sr-fresnoite.

The mean Sr—O distance in the title structure is 2.62 Å, which is comparible with the mean Sr—O distance of 2.61 Å in Sr_4_Ti_5_O_8_(Si_2_O_7_)_2_ (Miyajima *et al.*, 2002).

The O—Si—O angles deviate significantly from the ideal tetrahedral angle of 109.5°, indicating a strong distortion due to the presence of the TiO_5_ polyhedra in the titanosilicate layer.

Fig. 1 shows the Rietveld difference plot for the present refinement. The crystal structure of Sr_2_TiO(Si_2_O_7_) is displayed in Fig. 2 and consists of layers of corner-sharing SiO_4_ and TiO_5_ polyhedra extending parallel to the *ab* plane. These layers are separated along the *c* axis by layers of distorted SrO_6_ octahedra.

### Experimental

A synthetic sample of Sr-fresnoite was made by melting a stoichiometric mixture of SrCO_3_, TiO_2_ and SiO_2_ to form a glass. This glass was then quenched to 293 K, reground and then heated for 7 days at 1323 K. A small amount of CeO_2_ (*NIST* SRM 674*a*) standard was added to this powdered sample to act as an internal standard.

### Refinement

The powdered sample was loaded into a 0.7 mm diameter quartz capillary, prior to synchrotron X-ray powder diffraction data collection using the P02.1 high resolution powder diffraction beamline at the PETRA-III synchrotron. The beam on the sample was 0.8 mm wide and 1.27 mm high. Powder diffraction data were collected using a PerkinElmer XRD 1621 flat panel image plate detector, which was approximately 1.4 m from the sample. One powder diffraction dataset was collected at 293 K out to approx. 11.9°/2θ, the data collection time was 30 s. Powder diffraction data were converted to a list of 2θ and intensity using FIT2D (Hammersley *et al.*, 1996, Hammersley, 1997). Powder diffraction data in the range 1–11.7°/2θ were used for the Rietveld refinement. Data below 1°/2θ were excluded due to scatter from the beam stop and as there were no Bragg reflections in this region. Data above 11.7°/2θ were excluded as this corresponded to the edge of the image plate detector where the Bragg peaks were weaker.

The main Bragg reflections of the powder diffraction pattern could be indexed in space group *P*4*bm* with similar lattice parameters to those of PDF card 39–228 (ICDD, 1989). The unit cell of the incommensurately modulated structure (Höche *et al.*, 2002) corresponds to a doubled *c* axis compared to that given on the PDF card. The doubled *c* axis does not match with some of the low-angle Bragg reflections for the Sr_2_TiO(Si_2_O_7_) sample used in the present study, therefore this incommensurate structure was not used for Rietveld refinement. Bragg reflections for three impurity phases could also be identified in the powder diffraction data. SrTiO_3_ and SrSiO_3_ were formed as by-products during preparation.

Initial lattice parameters for the three Sr-containing phases were refined using local software. The CeO_2_ (*NIST* SRM 674*a*) standard was used to calibrate the sample to detector distance. The CeO_2_ lattice parameter was fixed at 5.4111 Å so as to calibrate the wavelength as 0.207549 Å.

The *P*4*bm* crystal structure of the mineral fresnoite (Ba_2_TiO(Si_2_O_7_); Ochi, 2006) was used as a starting model for the Rietveld refinement (Rietveld, 1969) of the structure of Sr_2_TiO(Si_2_O_7_). The crystal structures of SrSiO_3_ (Machida *et al.*, 1982), SrTiO_3_ (Mitchell *et al.*, 2000) and CeO_2_ (Goldschmidt & Thomassen, 1923) were used for the impurity phases in the refinement. Isotropic atomic displacement parameters were used for all phases. For the Sr_2_TiO(Si_2_O_7_) phase the Si—O and Ti—O distances in the SiO_4_ and TiO_5_ polyhedra were soft-constrained to those for Ba_2_TiO(Si_2_O_7_) (Ochi, 2006). The *U*_iso_ factors for all O sites were constrained to be the same. 81 (1) wt.% of Sr-fresnoite was present in this sample with 4.6 (3) wt.% CeO_2_, 7.0 (6) wt.% SrTiO_3_ and 7.4 (8) wt.% of SrSiO_3_ present as impurities.

### Crystal data

**Table:**

Sr_2_TiSi_2_O_8_	*D*_x_ = 3.890 (1) Mg m^−^^3^
*M**_r_* = 407.31	Synchrotron radiation, λ = 0.207549 Å
Tetragonal, *P*4*b**m*	µ = 0.43 mm^−^^1^
Hall symbol: P 4 -2ab	*T* = 293 K
*a* = 8.3200 (3) Å	Particle morphology: powder
*c* = 5.0239 (2) Å	white
*V* = 347.77 (2) Å^3^	cylinder, 20 × 0.7 mm
*Z* = 2	Specimen preparation: Prepared at 1323 K and 100 kPa

### Data collection

**Table:**

In-house design diffractometer	Data collection mode: transmission
Radiation source: Synchrotron	Scan method: continuous
Laue DCM diamond(111) & Si(111) monochromator	2θ_min_ = 0.053°, 2θ_max_ = 11.915°, 2θ_step_ = 0.008°
Specimen mounting: capillary	

### Refinement

**Table:**

*R*_p_ = 0.052	Excluded region(s): 0-1 and 11.7-12.0 degrees 2θ
*R*_wp_ = 0.073	Profile function: T-C-H Pseudo-Voigt function
*R*_exp_ = 0.031	71 parameters
*R*_Bragg_ = 0.093	5 restraints
χ^2^ = 31.068	
1476 data points	

### Fractional atomic coordinates and isotropic or equivalent isotropic displacement parameters (Å^2^)

**Table:**

	*x*	*y*	*z*	*U*_iso_*/*U*_eq_	
Sr1	0.3282 (2)	0.8282 (2)	0.017 (2)	0.0070 (8)*	
Ti1	0.00000	0.00000	0.558 (3)	0.007 (2)*	
Si1	0.1305 (6)	0.6305 (6)	0.535 (3)	0.019 (2)*	
O1	0.00000	0.50000	0.651 (5)	0.017 (3)*	
O2	0.1292 (15)	0.6292 (15)	0.191 (3)	0.017 (3)*	
O3	0.2985 (12)	0.5984 (15)	0.678 (3)	0.017 (3)*	
O4	0.00000	0.00000	0.209 (3)	0.017 (3)*	

### Geometric parameters (Å, º)

**Table:**

Sr1—O1^i^	2.733 (18)	Ti1—O3^viii^	1.961 (12)
Sr1—O2	2.499 (13)	Ti1—O3^ix^	1.961 (13)
Sr1—O2^ii^	2.676 (13)	Ti1—O4	1.75 (2)
Sr1—O2^iii^	2.676 (13)	Si1—O1	1.642 (11)
Sr1—O3^iv^	2.572 (15)	Si1—O2	1.73 (2)
Sr1—O3^v^	2.572 (14)	Si1—O3	1.594 (14)
Ti1—O3^vi^	1.961 (12)	Si1—O3^x^	1.594 (15)
Ti1—O3^vii^	1.961 (13)		
			
O3^xi^—Ti1^xii^—O3^xiii^	84.6 (9)	O3^x^—Ti1^xii^—O4^xii^	107.9 (12)
O3^xi^—Ti1^xii^—O3^xiv^	144.2 (10)	O3^xiv^—Ti1^xii^—O4^xii^	107.9 (12)
O3^xiv^—Ti1^xii^—O3^xiii^	84.6 (8)	O1—Si1—O2	110.3 (16)
O3^xi^—Ti1^xii^—O4^xii^	107.9 (12)	O1—Si1—O3^x^	108.0 (9)
O3^xiv^—Ti1^xii^—O3^x^	84.6 (8)	O1—Si1—O3	108.0 (10)
O3^xiii^—Ti1^xii^—O3^x^	144.2 (11)	O2—Si1—O3	117.1 (15)
O3^xiii^—Ti1^xii^—O4^xii^	107.9 (12)	O2—Si1—O3^x^	117.1 (15)
O3^x^—Ti1^xii^—O3^xi^	84.6 (9)	O3^x^—Si1—O3	95.2 (11)

Symmetry codes: (i) −*y*+1, *x*+1, *z*−1; (ii) −*y*+1, *x*+1, *z*; (iii) *y*, −*x*+1, *z*; (iv) *x*, *y*, *z*−1; (v) *y*−1/2, *x*+1/2, *z*−1; (vi) −*x*+1/2, *y*−1/2, *z*; (vii) *y*−1/2, *x*−1/2, *z*; (viii) *x*−1/2, −*y*+1/2, *z*; (ix) −*y*+1/2, −*x*+1/2, *z*; (x) *y*−1/2, *x*+1/2, *z*; (xi) −*x*+1/2, *y*+1/2, *z*; (xii) *x*, *y*+1, *z*; (xiii) −*y*+1/2, −*x*+3/2, *z*; (xiv) *x*−1/2, −*y*+3/2, *z*.
